# The respiratory microbiota during and following mechanical ventilation for respiratory infections in children

**DOI:** 10.1183/13993003.02652-2020

**Published:** 2021-04-01

**Authors:** Emma M. de Koff, Wing Ho Man, Marlies A. van Houten, Nicolaas J.G. Jansen, Kayleigh Arp, Raiza Hasrat, Elisabeth A.M. Sanders, Debby Bogaert

**Affiliations:** 1 Spaarne Academy, Spaarne Gasthuis, Hoofddorp and Haarlem, The Netherlands; 2 Dept of Paediatric Infectious Diseases and Immunology, Wilhelmina Children's Hospital and University Medical Centre Utrecht, Utrecht, The Netherlands; 3 Dept of Paediatrics, Willem-Alexander Children's Hospital and Leiden University Medical Centre, Leiden, The Netherlands; 4 Dept of Paediatrics, Spaarne Gasthuis, Hoofddorp and Haarlem, The Netherlands; 5 Dept of Paediatric Intensive Care, Wilhelmina Children's Hospital and University Medical Centre Utrecht, Utrecht, The Netherlands; 6 Dept of Paediatrics, Beatrix Children's Hospital, University Medical Centre Groningen, Groningen, The Netherlands; 7 Centre for Infectious Disease Control, National Institute for Public Health and the Environment, Bilthoven, The Netherlands; 8 Medical Research Council and University of Edinburgh Centre for Inflammation Research, Queen's Medical Research Institute, University of Edinburgh, Edinburgh, UK

## Abstract

The lower respiratory tract (LRT) harbours distinct, dynamic low-density microbial communities, established through micro-aspiration from the upper respiratory tract (URT) [1–3]. However, during intubation and mechanical ventilation, the endotracheal tube temporarily alters the anatomical continuity between URT and LRT, and may provide a bridge for airborne microbes and a barrier for micro-aspiration. Shortly after intubation for a severe LRT infection (LRTI) in children, the microbiota of the nasopharynx and LRT were shown to be very similar [4]. However, it remains unknown how the respiratory microbial community develops while the child recovers from the infection under treatment with mechanical ventilation and antibiotics. We therefore analysed respiratory microbiota changes in children participating in our study on acute LRTIs and who were admitted to the paediatric intensive care unit (PICU) for mechanical ventilation [4].

## Abstract 2

**During mechanical ventilation for an LRTI in children, the respiratory microbiota shifted from *Haemophilus-* and *Moraxella*-dominated profiles to profiles dominated by antibiotic-resistant *Enterobacteriaceae,* and *Staphylococcus* and *Streptococcus* species.** https://bit.ly/3pGfvhQ

*To the Editor*:

The lower respiratory tract (LRT) harbours distinct, dynamic low-density microbial communities, established through micro-aspiration from the upper respiratory tract (URT) [1–3]. However, during intubation and mechanical ventilation, the endotracheal tube temporarily alters the anatomical continuity between URT and LRT, and may provide a bridge for airborne microbes and a barrier for micro-aspiration. Shortly after intubation for a severe LRT infection (LRTI) in children, the microbiota of the nasopharynx and LRT were shown to be very similar [4]. However, it remains unknown how the respiratory microbial community develops while the child recovers from the infection under treatment with mechanical ventilation and antibiotics. We therefore analysed respiratory microbiota changes in children participating in our study on acute LRTIs and who were admitted to the paediatric intensive care unit (PICU) for mechanical ventilation [4].

The subset of 29 infants with community-acquired LRTI who required intubation and ventilation, was recruited between September 2013 and September 2016. The mean age of the cohort was 3.4 months (range 1.0–12.8 months) with 48% being female. All children were diagnosed with bronchiolitis. Conventional microbiological findings were available for 21 of the children. Antibiotics were administered to 28/29 children (25 co-amoxiclav, two cephalosporins, one azithromycin), five of whom were already started on treatment shortly before PICU admission. We obtained nasopharyngeal (NP) swabs, saliva and endotracheal aspirates (ETA) upon intubation (29 NP, 27 saliva, 25 ETA) and shortly before extubation (16 NP, 15 saliva, 14 ETA), which was mean±sd 5.9±2.6 days after intubation. Saliva was collected by placing an absorbent sponge in the cheek pouches and under the tongue until it became saturated with saliva, which was immediately transferred into glycerol DEPC medium using a sterile syringe. ETA was collected during routine suctioning of the endotracheal tube without instilling saline. We also obtained 20 NP swabs and 19 saliva samples during a follow-up visit, 51.9±13.5 days after PICU discharge.

Microbiota profiles were generated by sequencing of the 16S rRNA gene V4 hypervariable region. Sequence data was deposited in the NCBI Sequence Read Archive database (BioProject ID PRJNA669463). Methodological details have been previously published [4]. Overall, 29 NP, 27 saliva and 24 ETA samples at intubation, 12 NP, 14 saliva and 11 ETA samples at extubation, and 20 NP and 19 saliva samples at follow-up passed quality control (94.5% of available samples) and were eligible for further analysis. Infants with missing extubation samples were not significantly different from those with available samples in terms of baseline microbiota composition, age or gender (data not shown). Bacterial load was estimated by quantitative (q)PCR targeting the 16S rRNA gene [5, 6]. Pneumococcal presence and abundance was tested by *lytA* qPCR.

Alpha diversity was assessed using the Chao1 and Shannon indices for richness and diversity, respectively. Bacterial load and alpha diversity are summarised as median (interquartile range (IQR)), and differences by time point were evaluated using linear mixed-effect models including subject as a random effect. Differences in overall microbial composition were evaluated by permutational multivariate analysis of variance on the Bray–Curtis dissimilarity matrix with permutations constrained within subject. Microbiota clusters were assigned to each sample using unsupervised hierarchical clustering. Biomarker species of each cluster were identified using random forest classifier analysis, as previously described [7]. Associations between clusters and time points were tested with Fisher's exact tests. To assess microbiota concordance between niches, we calculated within-subject Bray–Curtis similarity (1 − Bray–Curtis dissimilarity), and Spearman's correlations between individual operational taxonomic unit (OTU) abundances.

Our results show that bacterial load dropped dramatically between intubation and extubation in all niches, though for saliva this difference was not significant (NP: from 92.2 (43.9–309.6) pg·µL^−1^ to 4.0 (1.6–24.0) pg·µL^−1^, p=0.024; saliva: from 270.3 (80.5–771.8) pg·µL^−1^ to 113.0 (20.1–290.6) pg·µL^−1^, p=0.158; ETA: from 126.8 (31.6–708.2) pg·µL^−1^ to 3.9 (2.8–13.4) pg·µL^−1^, p=0.039). After recovery, the bacterial load had increased only moderately in the NP (to 39.6 (13.9–144.6) pg·µL^−1^, p=0.459), and more strongly in saliva (to 364.8 (200.5–775.5) pg·µL^−1^, p=0.014). At the same time, richness and diversity remained comparable in the NP between intubation and extubation (Chao1: from 47.5 (34.6–62.3) to 46.8 (34.3–54.2), p=0.840; Shannon: from 1.17 (0.81–1.87) to 1.53 (0.81–1.75), p=0.945). In saliva, richness and diversity decreased between intubation and extubation, though the difference was only significant for diversity (Chao1: from 55.0 (43.1–62.6) to 46.8 (39.4–50.9), p=0.443; Shannon: from 2.1 (1.8–2.6) to 1.5 (0.9–1.8), p<0.001), which had also significantly increased again after recovery (to 2.3 (1.8–2.6), p<0.001). In ETA, we observed a modest nonsignificant increase in richness and diversity between intubation and extubation, which seemed mostly driven by an increase in evenness rather than species richness (Shannon: from 0.14 (0.07–0.76) to 0.99 (0.45–1.63), p=0.112; Chao1: from 32.8 (29.4–42.8) to 51.0 (40.5–62.3), p=0.065). Furthermore, the overall microbial community composition changed significantly between intubation and extubation in both NP (R^2^=5.8%, p<0.001) and saliva (R^2^=7.6%, p<0.001) and even more in ETA samples (R^2^=11.2%, p=0.002) (figure 1a–c). Consequently, when compared to recovery samples, the NP and saliva microbiota composition were even more different from the pre-extubation (NP: R^2^=12.8%, p=0.020; saliva: R^2^=10.2%, p=0.012) than from the intubation time point (NP: R^2^=7.0%, p=0.001; saliva: R^2^=3.5%, p=0.038), implying marked ecological impact and deviation from healthy microbiota as a consequence of antibiotic treatment and/or mechanical ventilation within a narrow timeframe.

**FIGURE 1:**
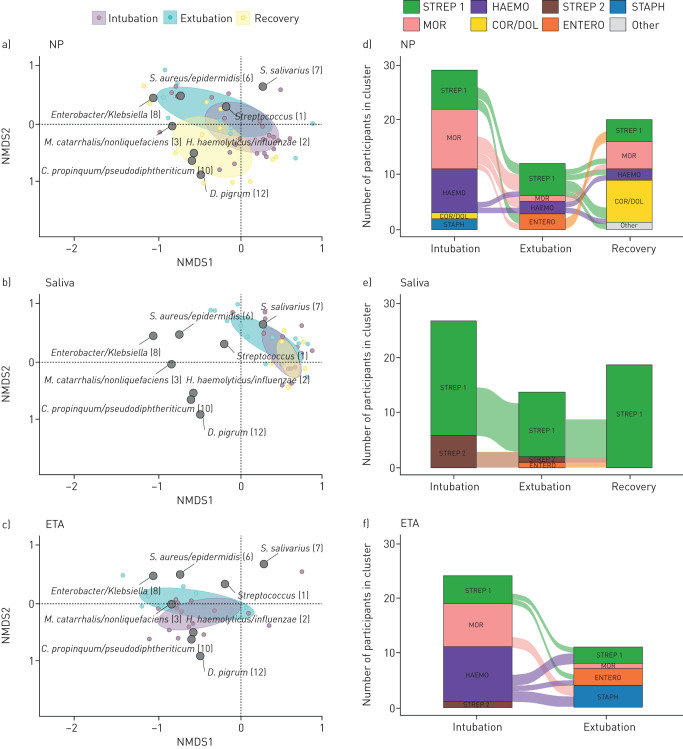
a–c) Nonmetric multidimensional scaling (NMDS) biplots based on the Bray–Curtis dissimilarity matrix visualising the overall microbiota composition in the a) nasopharynx (NP), b) saliva and c) endotracheal aspirate (ETA) at time of intubation, extubation and after 2 months recovery time, along with eight biomarker operational taxonomic units. Ellipses represent the standard deviation of the data points per subgroup. Alluvial plots of cluster transitions in the d) NP, e) saliva and f) ETA between time of intubation, extubation and after 2 months recovery time. Hierarchical clustering of all samples based on the Bray–Curtis dissimilarity matrix identified seven distinct clusters, characterised by either *Streptococcus (1)* (STREP 1), *Moraxella catarrhalis/nonliquefaciens* (MOR), *Haemophilus influenzae/haemolyticus* (HAEMO), *Corynebacterium propinquum/pseudodiphtheriticum* with *Dolosigranulum pigrum* (COR/DOL)*, Streptococcus salivarius (7)* (STREP 2)*, Enterobacter/Klebsiella* (ENTERO)*, or Staphylococcus aureus/epidermidis* (STAPH). Stacked bars represent the number of samples in each cluster per time point, and connections between bars represent transitions of participants with two consecutive samples available between time points.

We then performed clustering of NP, saliva and ETA microbiota profiles and distinguished seven clusters, characterised by either *Streptococcus (1)* (STREP 1), *Moraxella catarrhalis/nonliquefaciens* (MOR)*, Haemophilus influenzae/haemolyticus* (HAEMO)*, Corynebacterium propinquum/pseudodiphtheriticum* with *Dolosigranulum pigrum* (COR/DOL)*, Streptococcus salivarius (7)* (STREP 2)*, Enterobacter/Klebsiella* (ENTERO)*, or Staphylococcus aureus/epidermidis* (STAPH) (figure 1d–f). In NP and ETA, the MOR and HAEMO clusters predominated at intubation, and diminished following ventilation and antibiotic treatment. The COR/DOL profile was exclusively found in NP samples and mostly observed after recovery (p<0.05). At extubation, the MOR-cluster was only observed in the single infant who did not receive antibiotic treatment. By contrast, in saliva, both STREP 1 and STREP 2 clusters predominated at intubation, with the STREP 2 cluster diminishing at extubation, and being completely absent after recovery (not significant).

Overall, at extubation, the STREP 1, STAPH and ENTERO clusters were most prevalent, in line with expected changes following antibiotic exposure. Within the STREP 1 cluster, a shift from pneumococcal dominance at intubation to non-pneumococcal streptococci pre-extubation was observed (Spearman's correlation *lytA* Ct-values with *Streptococcus (1)* abundance at intubation: ρ=−0.68, p<0.001; at extubation: ρ=−0.08, p=0.883). Interestingly, the STAPH profile was only present in two NP samples at intubation, but predominated in ETA at extubation (p=0.006). The ENTERO cluster was uniquely found at extubation (p<0.05). *Enterobacter/Klebsiella* became the most predominant OTU in four children following (2–8 days of) intubation and ventilation (mean abundance NP: 50.8%, range 0.03–99.4%; saliva: 11.9%, range 0.0–45.1%; ETA: 64.5%, range 30.0–99.7%), even though this OTU was mostly absent at intubation, except for one child with a very low abundance in the NP of 0.008%. To identify this OTU at the species level, we attempted to re-culture the corresponding samples, and identified in three of those Gram-negative strains that were identified as *Enterobacter cloacae* by MALDI-TOF mass spectrometry. Together, these findings imply that the typically hospital-acquired and antibiotic-resistant pathobiont *E. cloacae* colonised and/or became dominant in the respiratory tract of these children during PICU stay. Similarly, we observed dominance of a *Stenotrophomonas* species (77.7% of ETA microbiota) in one case pre-extubation, despite it being nearly absent at intubation (0.002%), again suggesting selection or outgrowth during ventilation. In general, conventional culture performed at admission confirmed the predominant pathogens observed in the NP and/or ETA profile of 12/21 children. Culture results were negative in 5/21 children, and confirmation of non-predominant Gram positives but lack of detection of the predominant (Gram negative) pathogen was observed in four children. These findings underline that culture results, especially in children treated with antibiotics, often lack the ability to provide insight into presence and/or predominance of respiratory pathogens.

We previously demonstrated highly concordant NP and ETA microbiota at intubation in this cohort, suggesting the NP is the source community of the LRTI in young children [4]. However, interestingly, NP–ETA concordance at intubation (within-subject median Bray–Curtis similarity 0.66, IQR 0.44–0.81), had dropped pre-extubation (0.53, IQR 0.31–0.63), although this difference was not significant (Wilcoxon rank-sum test, p=0.188). Also, only 36 OTUs (combined relative abundance 36.6%) were still significantly correlated between NP and ETA samples pre-extubation, compared to 74 OTUs (combined relative abundance 84.2%) at intubation, suggesting non-NP microbes may have settled in the LRT community. We therefore investigated whether micro-aspiration could explain these findings, and studied the concordance between saliva and ETA samples both at intubation and pre-extubation. We observed that the concordance in microbial community composition between saliva and ETA was low at both intubation (within-subject median Bray–Curtis similarity 0.13, IQR 0.03–0.33), and pre-extubation (0.17, IQR 0.04–0.61). Moreover, the number of OTUs that correlated between both niches dropped from 70 OTUs at intubation (combined relative abundance 57.0%), to 52 OTUs at extubation (combined relative abundance 14.7%). Collectively, our data suggest that the NP is a more important source community for the LRT compared to the oral microbiota in children, and that NP, saliva and ETA microbiota evolve relatively independently during mechanical ventilation, which resulted in increased segregation between the URT and LRT microbial communities.

In summary, we observed that during intubation and ventilation, combined with antibiotic treatment in critically ill children suffering from a community-acquired LRTI, the respiratory microbiota composition clearly changed, even deviating further from “healthy” profiles. The bacterial load dropped and the relative abundance of predominant pathogens decreased, simultaneously allowing antibiotic-resistant bacteria, including *Staphylococcus* species, non-pneumococcal streptococci, and *Enterobacter/Klebsiella* species, to colonise and/or overgrow the respective niches. Furthermore, our data suggest differential effects of intubation/ventilation and/or antibiotic use on microbial communities in the respective niches. Our findings are in line with previous results in adults [8]. However, unlike findings in adults, the LRT microbiota of young children with a severe LRTI reflected the NP more than the oral microbiota before intubation, which did not change during ventilation. The main limitation of this study is its small sample size. Future, larger studies are required to disentangle independent effects of (different) antibiotic therapies and intubation for mechanical ventilation on respiratory microbiota dynamics. Further study is especially important because the respiratory microbiota composition during intubation has recently been related to clinical outcomes in adults. For instance, Dickson *et al.* [9] reported that detection of species of the Enterobacteriaceae family in the lungs of critically ill adults was associated with acute respiratory distress syndrome and prolonged duration of mechanical ventilation. Similarly, in a large cohort of mechanically ventilated patients, worse clinical outcomes were related to low alpha diversity combined with pathogen overgrowth in the LRT, in contrast to high alpha diversity with dominance of typically oral taxa [10]. In line with these findings, Woo *et al.* [11] observed that increased abundance of oral taxa including *Streptococcus* during intubation and ventilation were related to successful extubation. The findings presented here thus warrant similar studies of respiratory microbiota changes during intubation and ventilation in relation to recovery in critically ill paediatric patients, and exploration of methods to prevent rapid in-hospital acquisition and/or enrichment of antibiotic-resistant pathobionts in this already vulnerable patient population.

## Shareable PDF

10.1183/13993003.02652-2020.Shareable1 This one-page PDF can be shared freely online.
Shareable PDF ERJ-02652-2020.Shareable
