# Supplementary material for: The respiratory microbiota during and following mechanical ventilation for respiratory infections in children
Source: Eur Respir J. 2021 Apr 1;57(4):2002652. doi: 10.1183/13993003.02652-2020 (PMC8012590; doi:10.1183/13993003.02652-2020)
Supplement: Supplementary file 1 [file ERJ-02652-2020.Shareable.pdf]

AGORA
RESEARCH LETTER

SHAREABLE PDF

The respiratory microbiota during and following mechanical ventilation for respiratory infections in children

Emma M. de Koff[1,2], Wing Ho Man[1,3], Marlies A. van Houten[1,4], Nicolaas J. G. Jansen[5,6], Kayleigh Arp[2], Raiza Hasrat[2], Elisabeth A.M. Sanders[2,7] and Debby Bogaert[2,8]

Affiliations: [1]Spaarne Academy, Spaarne Gasthuis, Hoofddorp and Haarlem, The Netherlands. [2]Dept of Paediatric Infectious Diseases and Immunology, Wilhelmina Children's Hospital and University Medical Centre Utrecht, Utrecht, The Netherlands. [3]Dept of Paediatrics, Willem-Alexander Children's Hospital and Leiden University Medical Centre, Leiden, The Netherlands. [4]Dept of Paediatrics, Spaarne Gasthuis, Hoofddorp and Haarlem, The Netherlands. [5]Dept of Paediatric Intensive Care, Wilhelmina Children's Hospital and University Medical Centre Utrecht, Utrecht, The Netherlands. [6]Dept of Paediatrics, Beatrix Children's Hospital, University Medical Centre Groningen, Groningen, The Netherlands. [7]Centre for Infectious Disease Control, National Institute for Public Health and the Environment, Bilthoven, The Netherlands. [8]Medical Research Council and University of Edinburgh Centre for Inflammation Research, Queen's Medical Research Institute, University of Edinburgh, Edinburgh, UK.

Correspondence: Debby Bogaert, University of Edinburgh, QMRI, 47 Little France Crescent, Edinburgh, EH16 4TJ, UK. E-mail: d.bogaert@ed.ac.uk

@ERSpublications
During mechanical ventilation for an LRTI in children, the respiratory microbiota shifted from *Haemophilus*- and *Moraxella*-dominated profiles to profiles dominated by antibiotic-resistant *Enterobacteriaceae*, and *Staphylococcus* and *Streptococcus* species. https://bit.ly/3pGfvhQ

Cite this article as: de Koff EM, Man WH, van Houten MA, *et al*. The respiratory microbiota during and following mechanical ventilation for respiratory infections in children. *Eur Respir J* 2021; 57: 2002652 [https://doi.org/10.1183/13993003.02652-2020].

This single-page version can be shared freely online.

To the Editor:

The lower respiratory tract (LRT) harbours distinct, dynamic low-density microbial communities, established through micro-aspiration from the upper respiratory tract (URT) [1–3]. However, during intubation and mechanical ventilation, the endotracheal tube temporarily alters the anatomical continuity between URT and LRT, and may provide a bridge for airborne microbes and a barrier for micro-aspiration. Shortly after intubation for a severe LRT infection (LRTI) in children, the microbiota of the nasopharynx and LRT were shown to be very similar [4]. However, it remains unknown how the respiratory microbial community develops while the child recovers from the infection under treatment with mechanical ventilation and antibiotics. We therefore analysed respiratory microbiota changes in children participating in our study on acute LRTIs and who were admitted to the paediatric intensive care unit (PICU) for mechanical ventilation [4].

Copyright ©ERS 2021.. This version is distributed under the terms of the Creative Commons Attribution Licence 4.0.
